# Estimating Size and Trend of the North Interlake Woodland Caribou Population Using Fecal-DNA and Capture–Recapture Models

**DOI:** 10.1002/jwmg.380

**Published:** 2012-04-05

**Authors:** Peter N Hettinga, Arni Neil Arnason, Micheline Manseau, Dale Cross, Kent Whaley, Paul J Wilson

**Affiliations:** Natural Resources Institute, University of Manitoba70 Dysart Road, Winnipeg, MB, Canada R3T 2N2; Department of Computer Science, University of ManitobaWinnipeg, MB, Canada R3T 2N2; Western and Northern Service CentreParks Canada, 145 McDermot Avenue, Winnipeg, MB, Canada R38 0R9Natural Resources Institute, University of Manitoba70 Dysart Road, Winnipeg, MB, Canada R3T 2N2; Manitoba ConservationThe Pas, MB, Canada R9A 1M4; Manitoba ConservationThe Pas, MB, Canada R9A 1M4; Natural Resources DNA Profiling and Forensic Centre, Biology Department, Trent University2140 East Bank Drive, Peterborough, ON, Canada K9J 7B8

**Keywords:** abundance estimate, capture–recapture, fecal genotyping, genotyping error, non-invasive genetic sampling, *Rangifer tarandus caribou*, species at risk, trend analysis, woodland caribou

## Abstract

A critical step in recovery efforts for endangered and threatened species is the monitoring of population demographic parameters. As part of these efforts, we evaluated the use of fecal-DNA based capture–recapture methods to estimate population sizes and population rate of change for the North Interlake woodland caribou herd (*Rangifer tarandus caribou*), Manitoba, Canada. This herd is part of the boreal population of woodland caribou, listed as threatened under the federal Species at Risk Act (2003) and the provincial Manitoba Endangered Species Act (2006). Between 2004 and 2009 (9 surveys), we collected 1,080 fecal samples and identified 180 unique genotypes (102 females and 78 males). We used a robust design survey plan with 2 surveys in most years and analysed the data with Program MARK to estimate encounter rates (*p*), apparent survival rates (ϕ), rates of population change (λ), and population sizes (*N*). We estimated these demographic parameters for males and females and for 2 genetic clusters within the North Interlake. The population size estimates were larger for the Lower than the Upper North Interlake area and the proportion of males was lower in the Lower (33%) than the Upper North Interlake (49%). Population rate of change for the entire North Interlake area (2005–2009) using the robust design Pradel model was significantly <1.0 (λ = 0.90, 95% CI: 0.82–0.99) and varied between sex and area with the highest being for males in Lower North Interlake (λ = 0.98, 95% CI: 0.83–1.13) and the lowest being for females in Upper North Interlake (λ = 0.83, 95% CI: 0.69–0.97). The additivity of λ between sex and area is supported on the log scale and translates into males having a λ that is 0.09 greater than females and independent of sex, Lower North Interlake having a λ that is 0.06 greater than Upper North Interlake. Population estimates paralleled these declining trends, which correspond to trends observed in other fragmented populations of woodland caribou along the southern part of their range. The results of this study clearly demonstrate the applicability and success of non-invasive genetic sampling in monitoring populations of woodland caribou. © 2012 The Wildlife Society.

The non-invasive genetic sampling (NGS) of animal tissues using hair and fecal material provides a valuable source of DNA for use in wildlife research and monitoring (Taberlet et al. 1996, Kohn and Wayne 1997, Kendall and McKelvey 2008). In providing reliable information on the sex and identity of an animal, population demographic information can be obtained (Palsbøll et al. 1997, Kohn et al. 1999, Woods et al. 1999). NGS has been particularly successful in monitoring population sizes of black bear (*Ursus americanus*) and grizzly bear (*Ursus arctos*) using hair (Woods et al. 1999, Paetkau 2003) and fecal samples (Bellemain et al. 2005). The method has also been used on other species including coyotes (*Canis latrans*; Kohn et al. 1999), wolves (*Canis lupus*; Creel et al. 2003), badgers (*Meles meles*; Frantz et al. 2003), argali (*Ovis ammon*; Harris et al. 2010), Sitka black-tailed deer (*Odocoileus hemionus sitkensis*; Brinkman et al. 2011), capercaillie (*Tetrao urogallus*; Jacob et al. 2011) and mountain goat (*Oreamnos americanus*; Poole et al. 2011).

When using NGS as markers, consideration is given to ensure low genotyping error rates (Taberlet et al. 1996, Pompanon et al. 2005) and various methods have been proposed and tested to produce reliable genotypes and sex data (reviewed in Waits and Paetkau 2005). Commonly, samples are profiled and genotyped multiple times and the results of each run are compared until consensus is reached (Taberlet et al. 1997, Frantz et al. 2003, Pompanon et al. 2005). Other precautionary measures include improved laboratory procedures (Taberlet et al. 1996, Paetkau 2003, McKelvey and Schwartz 2004), handling and storage protocols (Piggott 2004, Roon et al. 2005*a*), and stringent survey protocols (i.e., based on season; Maudet et al. 2004). The absence of robust protocols to ensure correct genotyping information can result in costly laboratory work to mitigate poor sampling habits, the loss of data, and the misidentification of individual animals (Taberlet et al. 1996, McKelvey and Schwartz 2004, Roon et al. 2005*a*, *b*). The need for accurate genotyping information is particularly important when collecting genetic data for use in capture–recapture analysis as the inclusion of erroneous genotypes can result in overestimates of population size (Creel et al. 2003) or underestimates when insufficient number of loci are used to differentiate highly related individuals (Mills et al. 2000).

Ball et al. (2007) presented an improved method for non-invasive sampling where sloughed intestinal epithelial cells are wiped from the surface of fecal pellets and the amount of available target DNA is quantified to assess the quality of the samples. Using this method on winter collected fecal pellets, a large quantity of total DNA (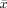 = 16.2 ng/µl) was obtained with a high concordance between total and target DNA estimates and only 10% of the samples showing relatively lower target-to-total DNA. High amplification rates have also been obtained from winter collected fecal pellets of other ungulate species including the Iberian ibex (*Capra ibex*), the Corsican mouflon (*Ovis mouflon*; Maudet et al. 2004), and the argali (Harris et al. 2010).

We studied 1 of the 57 local populations of the boreal ecotype of woodland caribou (*Rangifer tarandus caribou*; Environment Canada 2008), the North Interlake population, Manitoba, Canada. The boreal ecotype ranges from British Columbia to Newfoundland, Canada and is listed as threatened under the federal Species at Risk Act (2003) and provincial legislations including the Manitoba Endangered Species Act (2006). Generally, the western and more southerly located local populations are at greater risk of extirpation (Environment Canada 2008); the main factors being increased predation associated with natural or human-induced landscape conditions that favor greater densities of alternate prey species (Thomas and Gray 2002, McLoughlin et al. 2003). Accurate and precise population estimates are very difficult to obtain because the animals occur in low densities and in a clumped distribution over vast areas (Courtois et al. 2003). The main method used to estimate population sizes of woodland caribou consists of flying transect lines to identify caribou tracks followed by more intense search of these areas to count animals (Courtois et al. 2003). Radio-collaring of animals is required to assess and correct for visibility or to apply mark-resight calculations (Neal et al. 1993). Given the difficulties and errors associated with estimating population sizes, monitoring efforts primarily focus on trend data derived from the mean annual survival of radio-collared adult female and mean annual recruitment rate (Rettie and Messier 1998, McLoughlin et al. 2003).

To further the development of non-invasive methods, we used fecal DNA to estimate the size and trend of the North Interlake caribou population over a 5-year period (2005–2009). We used a robust design survey plan (Pollock 1982) with 2 surveys in most years and analyzed the data with Program MARK (White and Burnham 1999) to estimate encounter rates (*p*), apparent survival rates (ϕ), rates of population change (λ), and population sizes (*N*). We estimated these demographic parameters for males and females, and for 2 cryptic population genetic clusters identified by Ball et al. (2010) for the North Interlake area.

## STUDY AREA

**Figure 1:**
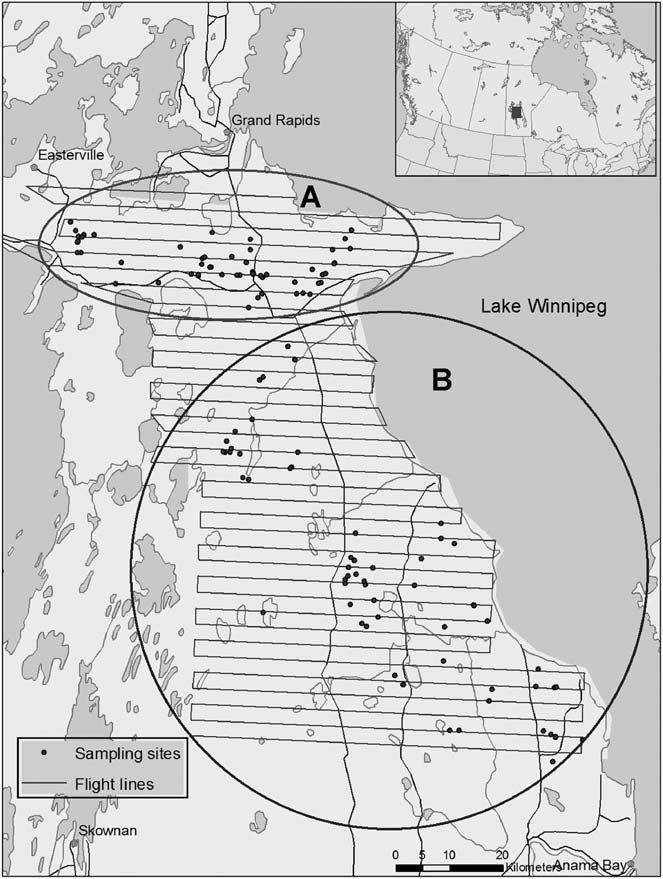
The North Interlake caribou range with flight lines flown at 3-km intervals, the 2004–2009 fecal pellet collection sites and the boundaries of the Upper (A) and Lower (B) North Interlake population genetic clusters.

The North Interlake region is part of the Mid-Boreal Lowland ecoregion (Ecological Stratification Working Group 1995) and lies between Lake Winnipeg and Lake Manitoba in central Manitoba, an area of approximately 4,000 km^2^ (Fig. 1). The ecoregion has a subhumid mid-boreal ecoclimate; defined by short, warm summers and long, cold winters. The mean summer temperature ranges from 15° C to 18.6° C and the mean winter temperature ranges from −19.7° C to −16° C; the mean annual rainfall is 362.4 mm and the mean annual snowfall is 111.5 cm (Environment Canada 2011). In most years, the period with sufficient snow cover on the ground allowing for tracking extends from the end of December to the end of March.

The landscape is topographically level to gently rolling, with an underlying geology comprised of dolomite limestone. The ecoregion is composed of mixed coniferous and deciduous forest, characterized by medium to tall closed stands of trembling aspen (*Populus tremuloides*) and balsam poplar (*P. balsamifera*) with white and black spruce (*Picea glauca* and *P. mariana*), and balsam fir (*Abies balsamea*) occurring in late successional stages. Jack pine (*Pinus banksiana*) stands are found on drier sites and extensive areas of treed muskegs (*P. Mariana* and tamarack, *Larix laricina*), fens, and bogs on poorly drained sites (Ecological Stratification Working Group 1995). Fire and drainage are the main factors influencing the vegetation pattern.

Other large mammal species and predators occurring in the area include white-tailed deer (*Odocoileus virginianus*), moose (*Alces alces*), wood bison (*Bison bison athabascae*), wolves, coyotes, and black bears. Two provincial highways dissect the area along with hydro transmission corridors and smaller roads and trails. The communities of Grand Rapids, Grand Rapids First Nation, Chemawawin First Nation, and Easterville are located just north of the study area. The area also corresponds to the southern component of a proposed National Park (Manseau et al. 2001).

Woodland caribou are found at low densities; the North Interlake caribou population has been estimated at 50**–**75 animals based on incidental animal and track observations (Manitoba Conservation 2006). Recently, Ball et al. (2010) examined the population genetic structure of woodland caribou in the region and, using individual-based clustering methods, identified 2 distinct genetic clusters in the North Interlake, herein referred to as Upper and Lower North Interlake. Ball et al. (2010) also documented more gene flow between Upper North Interlake and caribou ranges to the west (The Bog herd) than between Upper and Lower North Interlake. Similarly, recent landscape modeling analyses have shown limited connectivity of the North Interlake to other areas because of the large lakes to the east and west and hydro water reservoir to the north, and potential fragmentation between Upper and Lower North Interlake due to the road network (Fall et al. 2007, M. Manseau, Parks Canada, unpublished data).

## METHODS

### Sampling Design

We surveyed the study area systematically by fixed-wing aircraft in winter. The sampling period extended from 2004 to 2009 and consisted of 1 collection in 2004 (Mar, North Interlake only), 2005, and 2006 (Feb) and 2 collections in 2007 (Feb and Mar), 2008 (Jan and Mar), and 2009 (Jan and Feb). We flew transect lines 3 km apart and 3**–**4 days after a snowfall; this amounted to 2,200 km flown. Two observers recorded and mapped the location of tracks and cratering sites. Using a helicopter, a separate team of 3 people flew to those locations to collect fecal pellets. We completed each survey and the pellet collection within 2 days.

We established protocols to collect high quality samples. Typically, we collected a minimum of 10 pellets/sample and, to reduce the potential of collecting pellets from multiple animals in each sample, we selected pellets frozen together over single pellets. We collected the samples using disposable wooden sticks, placed in sterile bags with digitally produced labels and stored in a cooler onboard the aircraft.

At each cratering site, we collected approximately 1.4 times more samples than the number of woodland caribou thought to have been present. We froze all collected samples at −20° C and sent them to the Natural Resources DNA Forensics and Profiling Centre at Trent University in Peterborough, Ontario for genetic analysis.

### Genetic Analysis

In the laboratory, we thawed the samples and removed the mucosal coat surrounding fecal pellets for DNA analysis. Following quantification of 5 ng of target caribou DNA, we amplified the DNA using 9 polymorphic fluorescent-labeled microsatellite markers: RT5, RT6, RT9, RT24, BM888 (Wilson et al. 1997), Map2C, BM848 (Bishop et al. 1994), BMS1788, and RT7 (Cronin et al. 2005). For the sex identification, we used caribou-specific Zfx/Zfy primers. The protocol used to amplify the DNA is outlined in Ball et al. (2007).

Three persons independently genotyped the microsatellite DNA profiles following amplification. We scored peak alleles using GeneMarker® (SoftGenetics, State College, PA) with the assistance of documented scoring protocols that included details on expected allele peak morphologies for microsatellite loci amplified, expected strength of alleles in relative fluorescence units, and protocols for hard-to-score allele morphologies. Scoring results were entered in an online database where scores were compared and differences automatically flagged.

To determine the unique identification of genotypes and match samples across sampling times, we compared scores using GeneCap (Wilberg and Dreher 2004) and a probability of identity of full-sibs (*P*_sib_) cutoff of 0.05 (Woods et al. 1999). We reamplified samples identified as not having another genetic match after allowing for 1 or 2 allele mismatches. After the samples were reamplified, 3 persons scored them again independently following the same scoring protocols. We considered genotypes still differing by 1 or 2 alleles from another genotype as a match when calculated *P*_sib_ was ≤0.05. We gave samples not meeting this criterion a unique identification provided they had been successfully scored at a minimum of 6 loci.

### Error Rate

To determine genotyping error rate, we randomly selected a subset of 92 samples (9%) regardless of whether they were unique genotypes or replicate genotypes, reamplified them, and 3 persons scored them independently a second time. The per-locus error rate ranged from 0 to 0.013, approximating the recommended target per-locus rate by Roon et al. (2005*b*) of below 0.05 and ideally at 0.01. One-third of these errors resulted from heterozygote allele dropout with genotyping error accounting for the others. This low per-locus error rate can be attributed to the DNA template extracted from winter collected caribou pellets that is of sufficient quality for the quantification, normalization, and amplification of multiplex microsatellite profiles (Ball et al. 2007). To further minimize the inclusion of errors, we screened for replicate genotypes and reamplified all genotypes occurring only once in the dataset.

### Survey Design and Analysis

We followed a robust design (Pollock 1982) where a population is sampled for the purpose of calculating both open and closed population parameters. In using a robust design, all assumptions used in open and closed population models, as described below, apply (Williams et al. 2002). In our study, annual surveys were the open primary intervals, and the sampling times within each primary interval were the closed secondary intervals and took place over a relatively short-time frame. The robust design has a number of advantages, including allowance for capture heterogeneity within the closed secondary periods, the ability to efficiently estimate capture rates and resolve parameter identification problems in the open primary periods, and the ability to allow for temporary emigration (Williams et al. 2002). The robust design analyses in Program MARK require at least 2 secondary periods per primary period but the full set of heterogeneity models require a minimum of 3 secondary periods. This was beyond the survey resources available and would have strained the closure and encounter independence assumptions. Conducting 2 surveys within a 2-month period in each of 2007, 2008, and 2009, in addition to the single surveys in 2005 and 2006, approximated a robust design that conferred some of these advantages but was economically and logistically feasible yet still amenable to efficient analysis using robust design models.

### Mark-Recapture Analyses

Our goal was to use the data from 2005 to 2009 for both areas and sexes to model encounter rate (*p*) and apparent survival rate (ϕ) and then to estimate group-specific populations sizes (*N*) and rates of population change (λ). We use the terms encounter rate and capture rate interchangeably, although in an NGS context, encounter rate is the more appropriate term. We used a 2-stage analysis, starting with the Cormack–Jolly–Seber (CJS) models (Lebreton et al. 1992), to find appropriate model mechanisms for ϕ and *p*. Then we used the robust design models in MARK with Pradel models for the primary periods and closed captures models for the secondary periods. The robust design models produce estimates of all 4 parameter sets (*p*, ϕ, λ, and *N*), but it is much simpler to explore model mechanisms for *p* and ϕ first using the CJS models and then use a supported set of models from this stage as the basis for fitting the robust design models. This 2-stage approach was suggested by Arnason and Schwarz (1999) where it was demonstrated to be an effective way to decide on appropriate constraints on *p* and ϕ for POPAN models used to estimate population sizes and birth rates.

#### Cormack–Jolly–Seber modeling of ϕ and *p*

We constructed an 8-sample encounter history input file for Program MARK with 4 groups: Lower North Interlake, males and females and Upper North Interlake, males and females. By analyzing the 4 groups together, we were able to improve efficiency by finding common rates and mechanisms across groups.

We used the recaptures-only CJS models in MARK to explore group and covariate effects on ϕ and *p*. For ϕ, we allowed for the unequal time spacing δ_*t*_ between surveys using δ expressed in years so that all survival estimates were normalized to annual rates. We tested first if survival (per unit time) could be assumed time-constant within groups and then for group (sex and area) effects on ϕ by fitting all the survival models with the capture model *p*(*g* × *t*), which allows for group (*g*) and survey time (*t*) effects on *p*. We checked and adjusted the top-ranked models for correct parameter counts where confounding or estimates at the boundary required it. We ranked models using Akaike's Information Criterion adjusted for sample size (AIC_*c*_; Burnham and Anderson 2002). We then explored for similar temporal and group restrictions on *p* and, finding none, plotted *p* estimates from the top-ranked models to judge if *p* could be expressed as a linear function of sampling effort, as measured by the total number of sites sampled per survey, and the influence of group on the slope and intercept of this linear function. We then fit these (logit) linear functions, again with the top-ranked survival models. In total, we fit 17 models. We applied the RELEASE tests (Cooch and White 2011) as implemented in MARK to test for heterogeneity in capture or survival rates within each group.

The assumptions of the CJS models are 1) no loss of tags and proper identification of previously captured animals, 2) emigration of animals from the sampling area is permanent, 3) animals are independently sampled, 4) capture probability (*p*) at each survey is homogeneous across animals and, similarly, 5) apparent survival (ϕ) between each survey occasion is homogeneous across animals (Seber 1982, Williams et al. 2002). The CJS likelihood conditions on the numbers released and so homogeneity of rates does not strictly need to be extended to unmarked animals, but should ideally be the same for all animals regardless of age, encounter history, location, condition factor, or other individual traits or activity. A consequence of the conditioning is that the first capture rate, *p*_1_, cannot be estimated, but this is resolved later in the robust design analyses. To ensure no tag loss and accurate identification of unique genotypes (assumption 1), we adopted tight laboratory and scoring protocols. We considered the population to be closed to migration (assumption 2) based on earlier population genetic structure analyses (Ball et al. 2010). We addressed the third model assumption of the animals being independently sampled by allowing a sufficient amount of time between sampling occasions. The robust design models, discussed below, included models that permitted testing of sampling independence within years. Finally, we addressed the last model assumptions by accounting for heterogeneity due to group and covariate effects using model fits (Lebreton et al. 1992) and assessed any remaining within-group heterogeneity using the RELEASE tests.

#### Robust design models to estimate λ and *N*

We expanded the 8-sample encounter history input file to a 10-sample file with the addition of dummy samples (encoded by a dot in the encounter history) for the missing second secondary samples in 2005 and 2006. This ensured that MARK's requirement of at least 2 secondary samples per primary sample was met. When choosing the MARK data type, we used the *Pradel models including robust design* for the primary period models and *closed captures* for the secondary period models. The former models are based on Pradel (1996) and the latter on the closed models of Otis et al. (1978).

The net population rate of change between survey *t* and *t* + 1 is given by:


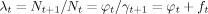


where γ_*t*+1_ is the seniority parameter (probability that an animal present at time *t* + 1 was also present at time *t*), and *f*_*t*_ is the per-capita recruitment rate defined as *B*_*t*_/*N*_*t*_ where *B*_*t*_ is the number of new recruits between time *t* and *t* + 1. The robust design analyses in MARK permit fitting of any of the parameterizations: (ϕ, *p*, λ), (ϕ, *p*, γ), or (ϕ, *p*, *f*) with group or covariate constraints on each parameter type. The first of these was used to fit time-constant λ models for each of the groups. Even if there are within- or among-year differences in λ, we were not interested in these short-term effects but in the overall trend as measured by time-constant λ models. Populations that follow a constant λ model (per unit time) will exhibit a log-linear trend in *N*_*t*_ versus *t* and we tested for this using the estimates of *N* as described below. We used time-constant models for each of 5 group-dependent sets: λ(·), λ(*a*), λ (*s*), λ(*a* × *s*), λ(*a* + *s*) where *a* designates the study site (area: Upper or Lower North Interlake) and *s* designates sex groups; λ(*a* × *s*) allows a different λ for each area and sex group, while the additive model λ(*a* + *s*) imposes a difference in λ between sex groups that is the same for each area, and vice versa. The constant model (same λ for each area and sex) is designated by λ(·). For each set, we fit a suite of 28 Pradel primary period models defined by all ϕ × *p* combinations of:


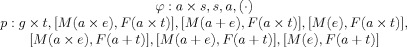


with different constraints being applied for males (M) and females (F). For males, where the CJS models indicated a strong response to effort (*e*; sites sampled), we applied (logit) linear models with different slopes and intercepts for each area (*a* × *e*), common slopes (*a* *+* *e*), or common slopes and intercepts for both areas (*e*). For females, where the effort response was weak, we allowed for area and time effects (*a* × *t*) or an additive area effect (*a* *+* *t*) where capture parameters vary with time but in parallel (logit scale) for the two areas. This suite included both unconstrained models and the constraints showing greatest support from the CJS analysis in stage 1. Within each set we used model averaging to account for model selection uncertainty (Burnham and Anderson 2002) when estimating the group-specific λ. We used a log link for λ and calculated the standard errors and 95% confidence intervals using the unconditional standard error estimates in MARK (White and Burnham 1999, Burnham and Anderson 2002). We estimated the population rate of change (λ) for the entire North Interlake area from set λ(·), the Upper and Lower North Interlake genetic clusters from set λ(*a*), the gender-specific rates from set λ(*s*), and the area- and sex-specific rates from the λ(*a* *+* *s*) and λ(*a* × *s*) sets combined.

We used closed capture models for the secondary periods. These include the models *M*_t_ and *M*_b_ from Otis et al. (1978). With 2 secondary samples, *M*_t_ is equivalent to a Lincoln–Petersen estimate and *M*_b_ allows the encounter rate at the second sample to differ between animals encountered versus not-encountered at the first sample time (the so-called behavioral response model). In MARK, the parameter *c* represents the encounter rate of animals that have been previously encountered. Constraining *c* = *p*_2_ reduces model *M*_b_ to model *M*_t_ and a likelihood ratio test of this constraint tests for behavioral heterogeneity. We used a likelihood ratio test both with constrained and unconstrained models for *p*, and included both constrained and unconstrained models for *c* in the model averaging. Earlier population genetic structure analyses (Ball et al. 2010) indicated the population groups were closed to immigration and the secondary intervals were likely closed to losses and additions by the season and spacing of the secondary samples (discussed further below).

The robust design model produces an estimate for *N* for each primary period in each area × sex group. These parameters are modeled in the likelihood and could be constrained but we did not impose constraints so as not to bias the λ estimates. As a check that the populations follow a log-linear trend (as implied by a time-constant λ model), we used the time-averaged 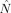 from the combined λ(*a* *+* *s*) and λ(*a* × *s*) sets to plot the log 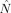 versus time along with the line with slope log λ constrained to pass through 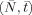, where 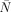 is the weighted average of the 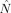 and 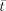 is the midpoint of the surveys. We used the weighted mean population sizes to calculate an overall sex ratio.

Assumptions of the Pradel model are the same as for the CJS model except that homogeneity of capture rate must extend to the unmarked animals as well. The ϕ and *p* estimates should be virtually identical to those of the CJS model with the same ϕ and *p* constraints if there is little heterogeneity in marked versus unmarked animals and if λ has not been over-constrained.

An alternate analysis for these data is to use the 8-sample encounter history file for the second stage analyses using Pradel models to estimate λ and POPAN models to estimate *N*, both averaged over the set of candidate *p* and ϕ models identified in the first stage CJS analyses. This more complex analysis produced estimates for all parameter sets that were virtually identical to those produced by the robust design analyses reported here, although the POPAN population estimates were more precise (see Table S1 and Fig. S1 for a summary of the POPAN results, available online at http://www.onlinelibrary.wiley.com). Compared to the robust design models, the POPAN models are more flexible in modeling closure assumptions (losses and new entries) but less flexible in modelling temporary emigration and capture heterogeneity.

## RESULTS

### Fecal Sampling, DNA Extraction, and Genotyping

**Figure 2:**
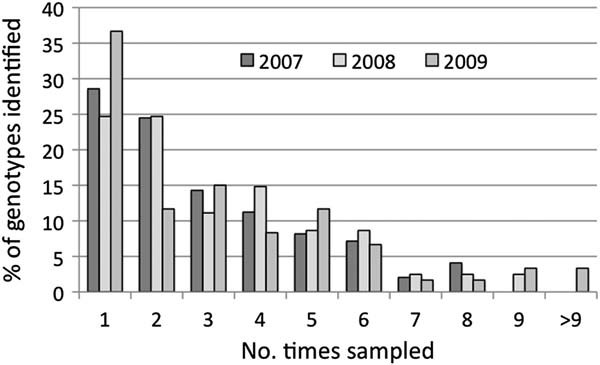
Percentage of unique genotypes of woodland caribou seen once or multiple times in the secondary sampling periods of the North Interlake area in 2007, 2008, and 2009.

**Figure 3:**
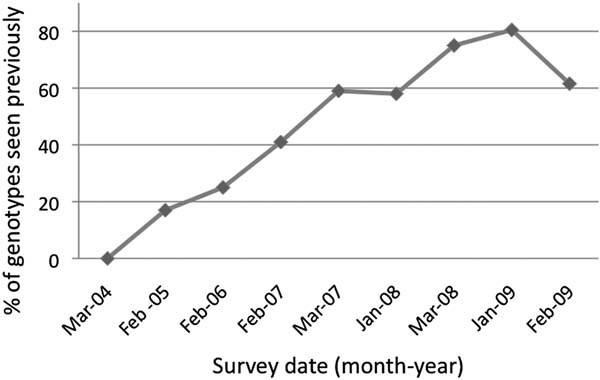
Percentage of unique genotypes of woodland caribou seen in previous surveys of the North Interlake area between 2004 and 2009.

Between 2004 and 2009, we collected 1,080 samples from 9 surveys, successfully genotyped 1,007 (93%) samples and obtained 180 unique multilocus genotypes (102 females and 78 males; Table 1). With the exception of the March 2004 collection, which only targeted the Upper North Interlake area, the number of sites sampled on each survey ranged between 8 (Feb 2009) and 17 (Feb 2007). We observed a large number of repeated observations within and between years; 34 unique genotypes (19%) were sampled on 1 occasion only. In a given year (2 surveys), 25–38% of the samples produced unique genotypes; other genotypes were observed multiple times (Fig. 2). The proportion of genotypes observed in previous surveys increased over time up to 80% in January 2009 (Fig. 3). The number of alleles ranged among the 9 loci from 9 (BM848) to 13 (BMS1788). Mean number of alleles per locus (Na) ranged from 7.6 to 9.1 and expected heterozygosity was 0.61 ± 0.02. The probability that 2 samples had identical genotypes (*P*_sib_) was low at 9.7 × 10^−4^ based on 9 loci and 7.3 × 10^−3^ based on 6 loci. For more information on levels of genetic diversity, see Ball et al. (2010).

**Table 1:** Sampling periods, number of sites and fecal pellet sampled, and number of unique genotypes obtained from 9 woodland caribou surveys in North Interlake, Manitoba, between 2004–2009

						Unique genotypes			
							
Year	Month	Days until next survey (δ)	Sites sampled	Samples collected	Samples scored	All	Males	Females	Samples scored per unique genotype
2004^a^	Mar	332	5	44	43	17	5	12	2.5
2005	Feb	375	10	87	85	32	16	16	2.7
2006	Feb	354	15	105	105	56	34	22	1.9
2007	Feb	34	17	182	165	73	37	36	2.3
	Mar	316	11	121	114	49	18	31	2.3
2008	Jan	40	15	127	118	55	19	36	2.1
	Mar	308	13	174	153	55	19	36	2.8
2009	Jan	29	11	134	118	41	11	30	3.0
	Feb		8	105	98	26	14	12	3.6
	Total		105	1,080	1,007	180	78	102	

a Only the Upper North Interlake area was sampled in 2004; results not used in the open and closed population models.

### Cormack–Jolly–Seber Models

We found strong support for time-constant survival across all years. All the models with time-varying survival rate were ranked low (14–17, Table 2) and had a ΔAIC_*c*_ in excess of 20. Support for a sex effect on survival was marginal, having less support than the ϕ(·) model with the same *p* model, whether highly constrained (Table 2, ranks 1 and 2) or unconstrained (rank 10 and 11). The ϕ(*s*) model had the greater likelihood (lower deviance), but received less support than the ϕ(·) model because of the additional parameter. The change in AIC was negligible in the case of the best-supported models (rank 1 and 2). The model-averaged estimates for ϕ from the robust design analysis show a sex effect with no area effect (Table 3) and are in agreement with the point estimates from the {ϕ(*s*) *p*[(M(*e*), F(*a* × *t*)]} CJS model (rank 2). The apparent support for an area effect in the rank 4 model (Table 2) is likely an artifact of the sex effect and an unequal sex ratio between the 2 areas.

**Table 2:** Cormack–Jolly–Seber models to fit survival rate (ϕ) and capture rate (*p*) of woodland caribou for the North Interlake area (2005–2009). All time-constant survival models (rank 1–13) are shown along with the least constrained time-varying model (rank 17). Model selection criteria include Akaike's Information Criterion corrected for sample size (AIC_*c*_), the difference in AIC_*c*_ relative to the top-ranked model (ΔAIC_*c*_), Akaike weights (*w*_*i*_), and number of parameters (*K*) for each model

Rank	Model	AIC_*c*_	ΔAIC_*c*_	*w*_*i*_	*K*
1	ϕ(·) *p*[M(*e*), F(*a* *×* *t*)]	849.7	0.0	0.31	17
2	ϕ(*s*) *p*[M(*e*), F(*a* *×* *t*)]	849.9	0.2	0.27	18
3	ϕ(·) *p*[M(*a* + *e*), F(*a* × *t*)]	850.2	0.5	0.24	19
4	ϕ(*a*) *p*[M(*e*), F(*a* × *t*)]	850.4	0.7	0.20	20
5	ϕ(*s*) *p*[M(*a* *+* *e*), F(*a* × *t*)]	850.6	1.0	0.17	21
6	ϕ(·) *p*[M(*a* × *e*), F(*a* × *t*)]	850.9	1.2	0.14	22
7	ϕ(*s*) *p*[M(*a* × *e*), F(*a* × *t*)]	854.2	4.5	0.03	20
8	ϕ(*g*) *p*[M(*e*), F(*a* × *t*)]	854.4	4.7	0.03	20
9	ϕ(*s*) *p*[M(*e*), F(*a* + *t*)]	855.8	6.1	0.01	12
10	ϕ(·) *p*(*g* × *t*)	867.3	17.6	0.00	29
11	ϕ(*s*) *p*(*g* × *t*)	867.9	18.2	0.00	30
12	ϕ(*a*) *p*(*g* × *t*)	869.5	19.8	0.00	30
13	ϕ(*g*) *p*(*g* × *t*)	872.5	22.8	0.00	32
17	ϕ(*g* × *t*) *p*(*g* × *t*)	903.6	53.9	0.00	52

*a*, area effect: parameter varies based on Upper vs. Lower North Interlake; *s*, sex effect; *g*, all 4 groups effect (*g* = *a* × *s*); *t*, time effect: parameter varies with survey time; (·), constant model; *a* × *e*, (logit) linear effort model with different slope and intercept for each area (*a*), or common slope (*a* *+* *e*), or common slope and intercept (*e*); [M(), F()] denotes separate constraints (specified in parentheses) applied to males and females.

**Table 3:** Apparent survival estimates (ϕ) of woodland caribou in North Interlake, Manitoba, between 2005–2009 from the best fitting Cormack–Jolly–Seber (CJS) sex-effect model^a^ and from model averaged estimates from the robust design models allowing for area and sex effects on rate of population change (λ) and ϕ

Area	Sex	Model	Survival	SE	CV (%)	95% CI
Lower and Upper North Interlake	Males	CJS	0.65	0.06	8.5	0.54–0.75
	Females		0.76	0.05	6.3	0.65–0.84
Lower North Interlake	Males	Robust design	0.65	0.06	8.7	0.53–0.75
	Females		0.74	0.05	6.6	0.64–0.83
Upper North Interlake	Males	Robust design	0.65	0.06	8.8	0.53–0.75
	Females		0.74	0.05	6.6	0.63–0.82

a ϕ(*s*) *p*[M(*e*), F(*a* × *t*)] has time constant survival rate (ϕ) with sex effect (*s*); capture probability (*p*) models differ for males (M) and females (F): male *p* is (logit) linear on effort (*e*) with the same slope and intercept for both areas (*a*); female *p* is area- and time-dependent.

**Figure 4:**
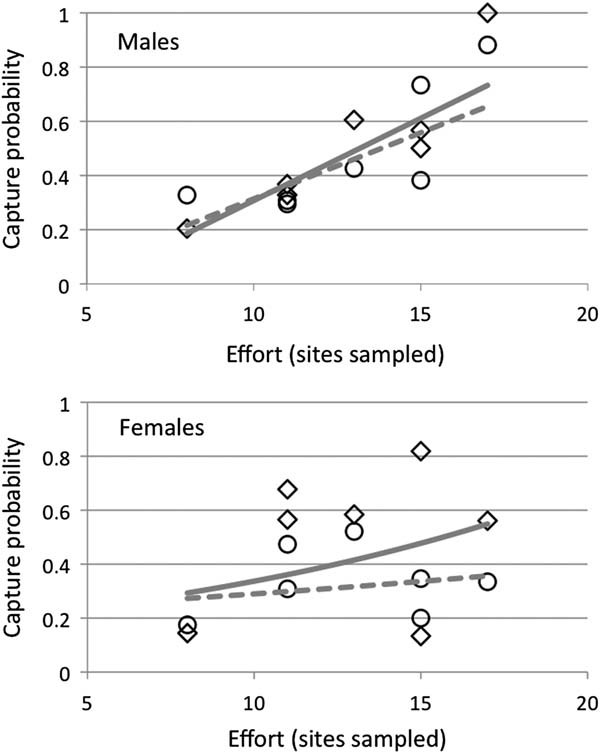
Plot of estimated capture probability of woodland caribou versus survey effort by sex and area in 2005–2009. Areas shown are Lower (○) and Upper North Interlake (◊). Points are from the [ϕ(*s*), *p*(*a* × *t*)] recaptures-only Cormack–Jolly–Seber (CJS) model: time-constant survival rate (ϕ) with sex effect (*s*); capture probability (*p*) with area (*a*) and survey time (*t*) effects. The fitted lines are from the {ϕ(*s*), *p*[M(*a* × *e)*, F(*a* × *e*)]} CJS effort model: male (M) and female (F) capture probability is logit linear on effort (*e*; sites sampled) with different slope and intercept for each sex and area. The solid line is Lower North Interlake and the dashed line is Upper North Interlake. The logit-linear effort model had high explanatory power for the males in both areas, but poor explanatory power for the females.

Capture rate appeared to operate according to different mechanisms for males and females. We found strong visual (Fig. 4) and model fitting support (Table 2) for a linear response of male (logit) capture rate to effort as measured by total sites sampled. The response was stronger to overall effort (sites sampled in both areas) than to the area-specific effort. A fortunate consequence of using a common effort covariate for both areas was that models with common slope or intercept or both could be tested and these were well supported by the model fits (Table 2). We included all 3 models in model averaging as none was clearly superior.

The female capture rates were not well explained by effort (Fig. 4) but a visual inspection of the data encouraged us to examine if additivity between areas was supported because, except for *t* = 1 (Feb 2006), the Upper North Interlake female capture rates were consistently higher at each *t* than the Lower North Interlake female capture rate. However, the hypothesis was not well supported; a likelihood ratio test of the rank 9 (additive) sub-model (Table 2) versus the rank 2 general model was rejected (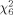 = 19.0, *P* = 0.004). The equivalent test using the robust design models gave a similar result (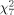 = 15.0, *P* = 0.035) Nevertheless, we included the additive model in the mix for model averaging in the robust design Pradel models, but they had little weight and so had little effect on the estimates.

### Rate of Population Change and Population Abundance

The apparent survival (ϕ) and capture probability (*p*) parameterizations in the top fitting robust design Pradel models were usually those of the top 2 ranked CJS models (Table 4). Model averaging did not add much to the variance of the estimated λ (Table 5) indicating that the λ estimates were somewhat robust to the model specified for ϕ and *p*. The ϕ and *p* estimates were virtually identical to those in the corresponding CJS model (Table 3) indicating no capture heterogeneity between marked and unmarked animals and that the time-constant λ models were not overly restrictive. The RELEASE tests raised no concerns about unexplained heterogeneity although sample sizes were only deemed sufficient for the largest, Lower North Interlake female, group. We found only marginal evidence for a behavioral response within years; a likelihood ratio test that *c* = *p*_2_ using robust design ϕ(*g*), *p*[M(*e*), F(*a* × *t*)], λ(*g*), *N*(*g* × *t*) as the base model was not significant (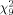 = 16.2, *P* = 0.06).

**Table 4:** Top 3 robust design models for each of 4 sets used with model averaging to estimate population net rate of change (λ) of woodland caribou for the North Interlake area (2005–2009). All top models had no recapture effects (c(session) = *p*_2_(session) for each primary session) and unconstrained population sizes *N*(*g* × *t*). Overall rank is model rank when all sets are combined. Model selection criteria include Akaike's Information Criterion corrected for sample size (AIC_*c*_), the difference in AIC_*c*_ relative to the top-ranked model (ΔAICc,), Akaike weights (*w*_*i*_), and number of parameters (*K*) for each model

Set	Rank	Overall rank	Model	AIC_*c*_	ΔAIC_*c*_	*w*_*i*_	*K*
λ(·)	1	1	ϕ(*s*) *p*[M(*e*), F(*a* × *t*)] λ(·)	299.3	0.0	0.54	41
	2	3	ϕ(*s*) *p*[M(*a* + *e*), F(*a* × *t*)] λ(·)	300.7	1.4	0.26	42
	3	6	ϕ(*s*) *p*[M(*a* *×* *e*), F(*a* × *t*)] λ(·)	302.4	3.1	0.11	43
λ(*a*)	1	4	ϕ(*s*) *p*[M(*e*), F(*a* × *t*)] λ(*a*)	301.0	0.0	0.58	42
	2	8	ϕ(*s*) *p*[M(*a* + *e*), F(*a* × *t*)] λ(*a*)	302.8	1.8	0.24	43
	3	12	ϕ(*s*) *p*[M(*a* *×* *e*), F(*a* × *t*)] λ(*a*)	304.4	3.4	0.10	44
λ(*s*)	1	2	ϕ(*s*) *p*[M(*e*), F(*a* × *t*)] λ(*s*)	300.6	0.0	0.55	42
	2	5	ϕ(*s*) *p*[M(*a* + *e*), F(*a* × *t*)] λ(*s*)	302.1	1.5	0.26	43
	3	9	ϕ(*s*) *p*[M(*a* *×* *e*), F(*a* × *t*)] λ(*s*)	303.8	3.2	0.11	44
λ(*a* × *s*)	1	7	ϕ(·) *p*[M(*e*), F(*a* × *t*)] λ(*a* *+* *s*)	302.5	0.0	0.43	43
	2	10	ϕ(*s*) *p*[M(*a* + *e*), F(*a* × *t*)] λ(*a* *+* *s*)	304.2	1.7	0.18	44
	3	13	ϕ(·) *p*[M(*e*), F(*a* × *t*)] λ(*a* × *s*)	304.6	2.1	0.15	44

ϕ, survival rate; *p*, capture rate; *a*, area effect, parameter varies based on Upper vs. Lower North Interlake; *s*, sex effect; *g*, all 4 groups effect (*g* = *a* × *s*); *t*, time effect, parameter varies based on survey time; (·), constant model; *a* × *e*, (logit) linear effort effect with different slope and intercept for each area group (*a*), or common slope (*a* *+* *e*), or common slope and intercept (*e*); [M(), F()] denotes separate constraints (specified in parentheses) applied to males and females.

**Table 5:** Rate of population change over 2005–2009 for the North Interlake woodland caribou population estimated by model averaging over the indicated model set. Variance indicates percent of estimated variance due to model averaging

			Rate of population change (λ)				
			
Area	Model set	Sex	Estimate	SE	Variance (%)	95% CI	CV (%)
North Interlake	λ(·)	Combined	0.90	0.04	0.5	0.82–0.99	4.9
	λ(*s*)	Males	0.95	0.06	0.1	0.83–1.07	6.6
	λ(*s*)	Females	0.85	0.06	0.1	0.73–0.97	7.3
Lower North Interlake	λ(*a*)	Combined	0.93	0.06	0.5	0.82–1.05	6.3
	λ(*a* × *s*)	Males	0.98	0.08	2.1	0.83–1.13	7.8
	λ(*a* × *s*)	Females	0.87	0.08	1.6	0.73–1.02	8.7
Upper North Interlake	λ(*a*)	Combined	0.87	0.06	0.7	0.75–0.98	6.7
	λ(*a* × *s*)	Males	0.91	0.08	2.8	0.75–1.06	8.6
	λ(*a* × *s*)	Females	0.83	0.07	2.1	0.69–0.97	8.7

*a*, area effect, parameter estimate varies based on Upper and Lower North Interlake; *s*, sex effect; (·), null model.

**Figure 5:**
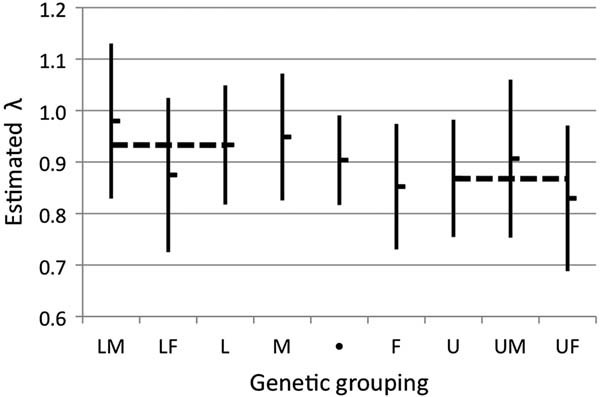
Estimates of rate of population change (λ) of woodland caribou, 2005–2009, plotted with 95% confidence intervals for various population groups: Lower North Interlake males and females (LM and LF), Upper North Interlake males and females (UM and UF), Lower and Upper North Interlake with sexes combined (L, U), males and females for both areas combined (M and F), and combined sexes and areas (·). Horizontal lines group area-specific estimates together. The additive effect of sex and area on λ can be seen in the first and last pairs of estimates. Estimates of λ are taken from the robust design model-averaged estimates.

**Figure 6:**
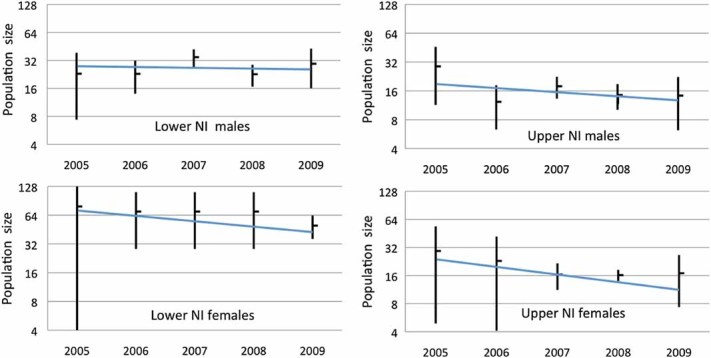
Population size estimates of woodland caribou in North Interlake (NI), 2005–2009, for each area-sex group plotted on a log scale versus survey year. Estimates with 95% confidence intervals are from the robust design *N* estimates model averaged over the set with sex and area effects on rate of population change (λ). Lines are the predicted (log linear) population trajectory using the fitted constant λ constrained to pass through the weighted population mean.

Some of the highest-ranking models had no sex or area effect on λ (models with overall ranks 1, 3, and 6 in Table 4). The model-averaged λ estimate over the λ(·) set was 0.90 and was significantly <1.0 (95% CI: 0.82–0.99). However, the best fitting models with both area and sex effects did not give significantly worse fits than the corresponding model with λ(·). For example, a likelihood ratio test of ϕ(*s*), *p*[M(*e*), F(*a* × *t*)], λ(*g*), *N*(*g* × *t*) versus the same model but with λ(·) (rank 1 model in Table 4) was not significant (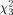 = 3.3, *P* = 0.35). The model averaged λ estimates from the λ(*a* × *s*) set (Table 5, Fig. 5) were below 1.0 for every area-sex cluster with the greatest being for Lower North Interlake males (λ = 0.98, 95% CI: 0.83–1.13) and the lowest for Upper North Interlake females (λ = 0.83, 95% CI: 0.69–0.97). This latter group was the only area-sex group whose λ was significantly less than 1. The additivity of λ between sex and area was supported on the log scale, and this translated roughly into males having a λ that was 0.09 greater than females and (independent of sex) Lower North Interlake having a λ that was 0.06 greater than Upper North Interlake. A population with a λ of 0.9 will decline by 50% in just over 7 years, a λ of 0.85 in 5 years, and a λ of 0.80 in just over 3 years. The population estimates (Fig. 6) also indicate declining trends at the rates estimated by λ. Using the weighted mean population sizes to compute an overall sex ratio gives 33% males in the Lower North Interlake and 49% in the Upper North Interlake area.

## DISCUSSION

### Survey Design and Assumptions

The robust design (Pollock 1982) is well suited for the assessment of populations using NGS. The use of secondary (within-year) closed surveys can provide estimates of abundance in the first year whereas open population models with annual surveys typically require 3 years before a full set of estimates can be formed. We did not do secondary sampling in 2005 and 2006 but would recommend starting secondary sampling in the first year of assessing a population. We were fortunate that a well-fitting effort model could be used to resolve identifiability of 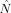 (at least for males) in 2005 but, in its absence, a complete robust design would have done the same. Heterogeneity concerns were seen to be resolved from the RELEASE tests, and this was likely because of removing the sex and area effects by stratifying by these clusters. Lack of handling likely removes all heterogeneity due to encounter history effects on capture and survival rates (which is what the RELEASE tests are sensitive to). Some heterogeneity due to age and activity levels may remain, but not enough to produce significant bias. Animals that travel and get sampled together tend not to have independent fates and this may have the effect of producing tighter confidence intervals around the estimates rather than biasing the estimates themselves (Williams et al. 2002). Careful consideration has to be given to spacing of the secondary samples. The minimum time between surveys was 1 month and this was adequate to allow a population to mix. This is supported by the continued rate of increase in sightings of new genotypes within years, at least in 2007 and 2008. Non-independence from too close a spacing would result in a tendency to re-encounter the same animals and would produce underestimates of abundance. Surveys should not be too far apart because of the limited winter survey season and the risk of non-closure (addressed below).

Using NGS with capture–recapture models avoids problems associated with tag loss where a marked animal may lose its tag or have its tag misread. However, some consideration was given to the possibility of adding animals into a population through the creation of false genotypes, which introduce bias of the *N* estimates (Mills et al. 2000, Waits and Leberg 2000, Creel et al. 2003). The amplification and scoring protocols used in this study were robust and many steps were taken to ensure the proper identification of unique genotypes. Those included the initial quantification of available DNA with winter-collected samples (Ball et al. 2007), the reamplification of genotypes that only appeared once, the reamplication of samples with more than 1 missing loci, the scoring of unique genotypes by multiple independent scorers, and the use of an online database to facilitate the comparison of results (Galpern et al. 2012).

The sampling design used in this study allowed for the analysis of various sampling groups based on sampled genetic characteristics. This presented a number of advantages for management in being able to assess overall sampling success and monitoring options (Mulders et al. 2007, Harris et al. 2010). The division of samples based on sex has revealed biased sex ratios and the need for calculating sex-based population estimates and trends (Boulanger et al. 2004, Mulders et al. 2007). In estimating population sizes of ungulate populations, this is particularly relevant as population growth rates are largely determined by the survival rate of adult females (Gaillard et al. 1998). In treating genetic clusters as groups in MARK, we were able to obtain parameters for the Upper North Interlake separately. Because of the Upper North Interlake population's smaller size, a relatively fragmented range, and the propensity of inverse density dependence effects (Wittmer et al. 2005), being able to assess population parameters for this group separately provided additional information. Precision of the estimated rates and abundance for the Upper North Interlake was greatly enhanced, relative to an analysis of the encounter histories on their own, by deriving these as part of a combined analysis using groups because the Upper North Interlake showed commonalities of sex-specific survival, capture, and population change rates with the larger Lower North Interlake.

### Population Closure Assumptions

When geographic area is treated as a population grouping for analysis, each area population should represent a well-defined target population, not subject to temporary emigration and boundary effects or population interchange. Population genetic structure analysis and landscape modeling for the greater region revealed a significant level of fragmentation between the North Interlake population and neighboring populations and within the North Interlake area (Ball et al. 2010). Also, out of 1,007 samples genotyped between 2004 and 2009, only 2 genotypes were captured in both the Upper and Lower North Interlake and this occurred during the same survey (Feb 2006); we suspect this may have been caused by a labeling error (sample nos. 2406 and 2409). This lack of movement between Upper and Lower North Interlake may be due to the intersection of 2 major highways, hydro transmission lines, and smaller roads and trails that affect animal movements (P. Galpern, University of Manitoba, unpublished data). Movement between the Upper North Interlake animals and the neighboring populations remains a possibility (Ball et al. 2010); although it is considered limited, particularly between survey periods used to estimate *N*.

A small possibility exists that some animals may have died during the 1-month period between within-year surveys; therefore, they would have become unavailable to sampling. However, research on woodland caribou has shown very low woodland caribou mortality rates in January and February relative to other months (Seip 1992, McLoughlin et al. 2003, Arsenault and Manseau 2011). In the POPAN analysis, we constrained new entries to 0, but used time-constant survival models that allowed for small losses during the secondary periods. This produced similar results to the robust design model that imposes strict closure within primary periods. For the annual survey intervals, apparent survival is indicative of losses to the population through both deaths and emigration with the assumption that survival is the same for all animals. Little movement occurs to and from our sampling area (Ball et al. 2010); therefore, a large part of this assumption is being met a priori and estimates of apparent survival should closely match actual survival.

### Trend in Abundance

Despite the lack of adherence to a strict robust design, we were able to analyze the data for population trend using the Pradel models within the robust design framework with allowance for groups and unequal sampling intervals. By adopting time-constant λ models in the Pradel formulation, we were able to estimate long-term trends over the complete 4-year interval (2005–2009), ignoring within- and among-year variations. We confirmed that these variations were unsystematic about the long-term trend by plotting the population estimates and confirming that populations followed the predicted log-linear trend (Fig. 6); otherwise, the time-constant λ estimates would be misleading. It is also important to use model averaging as there were many competing CJS models for *p* and ϕ that had almost equal weight and trend estimates (Arnold 2010).

The results of the Pradel model estimates for the period of 2005–2009 (Fig. 5) indicated a declining trend of 0.90 for the North Interlake population as whole, a declining trend for females, and non significant results for males. We found support for different trends for males and females, in both Upper and Lower North Interlake genetic clusters. The λ estimates have the same ranking by group area as the ranking of their population size; the smallest populations (Upper North Interlake) had the greatest rates of decline. The declining trend is within the range of estimates obtained from 9 populations of woodland caribou in Alberta (λ: 0.88–1.03; Alberta Woodland Caribou Recovery Team 2005) and 17 populations in British Columbia (λ: 0.82–1.03; Wittmer et al. 2005), pointing to a pattern for local populations occurring at the southern limit of the species range.

## MANAGEMENT IMPLICATIONS

The capture–recapture NGS method used in this study provides both population size and trend estimates. It also generates ancillary data for population genetic structure analysis (Ball et al. 2010) and landscape genetic analysis (Galpern et al. 2011) that can detect early signs of fragmentation, decline, or other information pertaining to biological and ecological processes (Bruggeman et al. 2010). The robust design model, using annual primary periods, normalizes survival and trend rates to annual rates and estimates them efficiently. It is a powerful tool for detecting rates of decline; with fewer than 2 surveys per year, we detected a 10% decline within 5 years. Stetz et al. (2010) used simulations in MARK to show that a robust design with 2 surveys per year was adequate to detect a 3% decline within 6 years using typical bear-rub survey methods. These results clearly show that non-invasive sampling combined with good survey design and careful genetic and capture–recapture analysis provides a powerful and robust means of monitoring wildlife populations.
