# Supplementary figures and images for: Estimating Size and Trend of the North Interlake Woodland Caribou Population Using Fecal-DNA and Capture–Recapture Models
Source: J Wildl Manage. 2012 Apr 5;76(6):1153–64. doi: 10.1002/jwmg.380 (PMC3437481; doi:10.1002/jwmg.380)

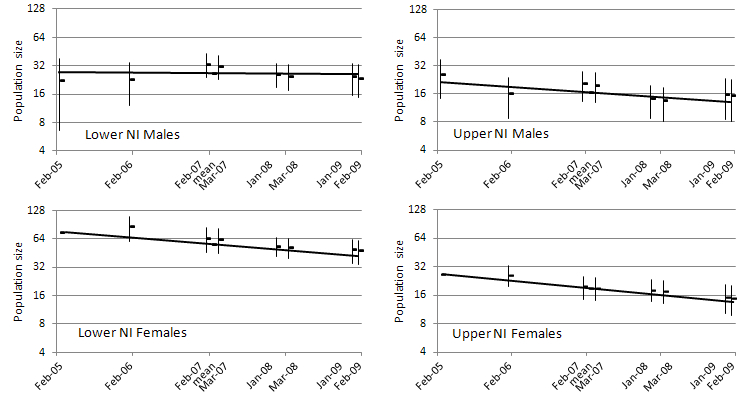

Supplement: Supplementary file 1 [file jwmg0076-1153-SD1.jpg]
