# Supplementary material for: Estimating Size and Trend of the North Interlake Woodland Caribou Population Using Fecal-DNA and Capture–Recapture Models
Source: J Wildl Manage. 2012 Apr 5;76(6):1153–64. doi: 10.1002/jwmg.380 (PMC3437481; doi:10.1002/jwmg.380)
Supplement: Supplementary file 2 [file jwmg0076-1153-SD2.doc]

**List of Figure Titles:**

**Figure S1.** Population size estimates for each area-sex group plotted on a log scale versus survey date. Estimates with 95% CI are from the POPAN (Jolly-Seber) model φ(*s*), *p*{M(*e*), F(*a* × *t*)}, *pent*(*a* × *s* × *t*). Lines are the predicted (log linear) population trajectory using the fitted constant λ estimates from the corresponding Pradel model φ(*s*), *p*{M(*e*), F(*a* × *t*)}, λ(*a* × *s*) reported in Table 1S constrained to pass through the weighted population mean. There are no estimates of *N* for the first survey for females due to parameter non-identifiability; the identifiability is resolved for male groups by the effort model.

**Table S1.** Estimates (EST) with standard errors (SE), coefficient of variation (CV), and 95% confidence interval (CI) for male (M) and female (F) woodland caribou in the Lower (L) and Upper (U) North Interlake area (2005−2009) compared using the Robust Design vs the POPAN/Pradel models. All are unaveraged estimates using the φ(*s*), *p*{M(*e*), F(*a* × *t*)} survival/capture model. The Robust Design and Pradel models use λ(*a* × *s*) and the POPAN model uses *pent*(*a* × *s* × *t*). The Robust Design produces estimates of survival (φ), population rate of change (λ) for each area and sex group and a population estimate (*N*) for each primary period (year) assuming that secondary surveys (within years) are closed. The POPAN model provides the estimates of φ on the right side of the table and an estimate of *N* for every secondary survey assuming that there are no births/new entries within years. Some POPAN estimates are unidentifiable (U) due to parameter confounding. The estimates of λ (right side) are from the Pradel model.

|  | **Robust Design** | | | |  | **POPAN/Pradel** | | | |
| --- | --- | --- | --- | --- | --- | --- | --- | --- | --- |
| **Group /**  **Time** | **EST** | **SE** | **CV%** | **95% CI** |  | **EST** | **SE** | **CV%** | **95% CI** |
|  |  |  |  |  | **(φ)** |  |  |  |  |
| LM | 0.65 | 0.05 | 8.4 | (0.53, 0.74) |  | 0.64 | 0.05 | 8.4 | (0.53, 0.74) |
| LF | 0.74 | 0.05 | 6.3 | (0.64, 0.82) |  | 0.76 | 0.05 | 6.2 | (0.65, 0.84) |
| UM | 0.65 | 0.05 | 8.4 | (0.53, 0.74) |  | 0.64 | 0.05 | 8.4 | (0.53, 0.74) |
| UF | 0.74 | 0.05 | 6.3 | (0.64, 0.82) |  | 0.76 | 0.05 | 6.2 | (0.65, 0.84) |
|  |  |  |  |  | **(λ)** |  |  |  |  |
| LM | 1.00 | 0.084 | 8.4 | (0.85, 1.18) |  | 0.99 | 0.081 | 8.2 | (0.84, 1.16) |
| LF | 0.86 | 0.078 | 9.1 | (0.72, 1.03) |  | 0.86 | 0.078 | 9.0 | (0.72, 1.03) |
| UM | 0.88 | 0.083 | 9.4 | (0.73, 1.06) |  | 0.88 | 0.081 | 9.2 | (0.74, 1.05) |
| UF | 0.84 | 0.079 | 9.4 | (0.70, 1.02) |  | 0.84 | 0.077 | 9.2 | (0.70, 0.99) |
|  |  |  |  |  | **(*N*)** |  |  |  |  |
| LM 2005 | 22.0 | 7.2 | 32.9 | (13.4, 44.3) |  | 22.3 | 8.1 | 36.1 | (6.52, 38.1) |
| 2006 | 22.5 | 4.3 | 19.0 | (17.4, 35.5) |  | 23.1 | 5.6 | 24.2 | (12.1, 34.1) |
| 2007 | 34.3 | 3.5 | 10.1 | (30.3, 45.3) |  | 33.2 | 4.8 | 14.3 | (23.8, 42.5) |
|  |  |  |  |  |  | 31.9 | 4.7 | 14.6 | (22.7, 41.0) |
| 2008 | 22.4 | 2.8 | 12.7 | (19.4, 32.0) |  | 26.2 | 3.9 | 14.9 | (18.5, 33.8) |
|  |  |  |  |  |  | 25.0 | 3.9 | 15.5 | (17.3, 32.5) |
| 2009 | 28.4 | 6.1 | 21.6 | (20.7, 46.5) |  | 24.8 | 4.8 | 19.2 | (15.4, 34.0) |
|  |  |  |  |  |  | 23.9 | 4.7 | 19.6 | (14.7, 33.1) |
| LF 2005 | 83.0 | 56.0 | 67.5 | (27.0, 281.0) |  | U | U |  |  |
| 2006 | 72.2 | 22.4 | 31.0 | (43.8, 137.0) |  | 85.3 | 13.3 | 15.5 | (59.2, 111.) |
| 2007 | 67.7 | 11.0 | 16.3 | (53.1, 98.6) |  | 65.0 | 10.0 | 15.4 | (45.3, 84.6) |
|  |  |  |  |  |  | 63.4 | 9.9 | 15.6 | (44.0, 82.6) |
| 2008 | 49.8 | 7.0 | 14.0 | (41.2, 70.5) |  | 53.1 | 6.2 | 11.6 | (41.0, 65.1) |
|  |  |  |  |  |  | 51.5 | 6.2 | 12.0 | (39.3, 63.5) |
| 2009 | 43.7 | 9.2 | 21.1 | (32.8, 72.2) |  | 48.8 | 7.1 | 14.6 | (34.8, 62.7) |
|  |  |  |  |  |  | 47.7 | 7.1 | 15.0 | (33.7, 61.7) |
| UM 2005 | 30.5 | 8.8 | 29.0 | (19.3, 56.4) |  | 25.6 | 5.7 | 22.4 | (14.3, 36.8) |
| 2006 | 12.6 | 3.1 | 24.5 | (9.4, 23.2) |  | 16.3 | 3.9 | 23.9 | (8.65, 23.8) |
| 2007 | 18.1 | 2.4 | 13.0 | (15.8, 26.7) |  | 20.5 | 3.6 | 17.8 | (13.3, 27.6) |
|  |  |  |  |  |  | 19.7 | 3.6 | 18.1 | (12.7, 26.7) |
| 2008 | 14.8 | 2.2 | 15.1 | (12.7, 23.1) |  | 14.1 | 2.8 | 19.5 | (8.73, 19.5) |
|  |  |  |  |  |  | 13.5 | 2.7 | 20.1 | (8.16, 18.7) |
| 2009 | 14.9 | 4.2 | 28.0 | (10.3, 28.6) |  | 15.9 | 3.8 | 24.1 | (8.38, 23.4) |
|  |  |  |  |  |  | 15.3 | 3.8 | 24.5 | (7.98, 22.7) |
| UF 2005 | 28.1 | 12.1 | 43.2 | (15.9, 70.7) |  | U | U |  |  |
| 2006 | 22.4 | 9.4 | 41.9 | (12.9, 55.2) |  | 26.0 | 3.3 | 12.8 | (19.5, 32.5) |
| 2007 | 16.3 | 2.6 | 15.8 | (14.4, 27.5) |  | 19.8 | 2.8 | 14.0 | (14.4, 25.2) |
|  |  |  |  |  |  | 19.3 | 2.8 | 14.2 | (13.9, 24.7) |
| 2008 | 16.2 | 1.1 | 7.0 | (16.0, 23.9) |  | 18.4 | 2.4 | 13.1 | (13.6, 23.1) |
|  |  |  |  |  |  | 17.8 | 2.4 | 13.6 | (13.0, 22.5) |
| 2009 | 17.1 | 5.0 | 29.1 | (12.5, 35.7) |  | 15.4 | 2.7 | 17.5 | (10.1, 20.7) |
|  |  |  |  |  |  | 15.1 | 2.7 | 17.9 | (9.79, 20.3) |
